# Early Diagnosis Value of DCE-MRI Hemodynamic Parameters in Hepatocellular Carcinoma

**DOI:** 10.1155/2022/9556589

**Published:** 2022-05-13

**Authors:** Xixi Mu, Yue Zhong, Xuan Zhang, Changjun Qu

**Affiliations:** ^1^Department of General Surgery, Xi'an Central Hospital, Xi'an 710003, Shaanxi Province, China; ^2^Department of General Surgery, The Second People's Hospital of Shaanxi Province, Xi'an 710005, Shaanxi Province, China; ^3^Department of Hepatobiliary Surgery, Xijing Hospital, The Fourth Military Medical University, Xi'an 710032, Shaanxi Province, China; ^4^Department of Radiology, Xi'an Central Hospital of Medical College of Xi'an Jiaotong University, Xi'an 710003, Shaanxi Province, China

## Abstract

**Objective:**

To probe into the early diagnosis value of transfer coefficient (*K*^trans^) and rate constant (*K*_ep_) of the dynamic contrast-enhanced magnetic resonance imaging (DCE-MRI) hemodynamic parameters in patients with hepatocellular carcinoma (HCC).

**Methods:**

Fifty patients with HCC diagnosed in our hospital from May 2017 to May 2020 were studied retrospectively as the patient group (PG), and 45 cases with benign liver nodules diagnosed in our hospital during the same period were collected as the control group (CG). *K*^trans^, *K*_ep_, and AFP levels were compared between PG and CG. The diagnostic value of *K*^trans^ and *K*_ep_ in HCC were observed, and their correlations with patient clinical data were analyzed. The diagnostic value of *K*^trans^ and *K*_ep_ combined with AFP in clinical staging, differentiation degree, and distant metastasis was compared.

**Results:**

*K* ^trans^, *K*_ep_, and AFP were notably higher in cases than in controls (*P* < 0.05), indicating their high diagnostic value in HCC. *K*^trans^ and *K*_ep_ present elevated expression in HCC patients with clinical stage III-IV, low differentiation, and distant metastasis (*P* < 0.05). Furthermore, it is found that the combined detection with either *K*^trans^ or *K*_ep_ can improve the clinical diagnostic value of AFP in the clinical stage and differentiation of HCC. However, the combined diagnosis showed little effect in raising the sensitivity of AFP in distant metastasis.

**Conclusion:**

DCE-MRI hemodynamic parameters *K*^trans^ and *K*_ep_ have high clinical value in early diagnosis and differentiation of HCC patients.

## 1. Introduction

Hepatocellular carcinoma (HCC), the sixth most common cancer globally as well as the second leading cause of cancer-related mortality, is the most commonly seen primary malignant tumor of the liver [1, 2]. HCC cases and deaths in China account for more than 50% of the global total [3]. The pathogenesis of HCC is heterogeneous and complex [4]. Current investigations indicate that approximately 70%-90% of HCC develops in the context of established cirrhosis or advanced fibrosis [5]. Due to the nonspecific early symptoms as the onset of HCC is hidden, the rate of missed diagnosis and misdiagnosis is high [6]. Consequently, the disease has progressed to the advanced stage when patients come to see a doctor with clinical symptoms [7]. Currently, the overall prognosis of HCC is still poor with a 5-year survival rate of less than 10%, except for some patients who were diagnosed early and underwent surgical resection or liver transplantation in time [8].

In recent decades, noninvasive imaging modalities, including ultrasound (US), computed tomography (CT), and magnetic resonance imaging (MRI), have played a key role in evaluating HCC [9]. Unlike most solid cancers, HCC can be diagnosed by nonhistological evidence [10]. Clinically, noninvasive imaging techniques such as CT or MRI are often used to diagnose and stage HCC [11]. Compared with traditional contrast-enhanced CT, MRI has the advantages of high sensitivity and spatial resolution, as well as enhanced soft tissue contrast and no radiation damage [12]. While based on the gradient echo T1W1 sequence of three-dimensional volumetric thin-layer scanning, multiphase dynamic contrast-enhanced magnetic resonance imaging (DCE-MRI) injects magnetic resonance contrast agent into the vein mass to carry out repeated, multiphase, and rapid scanning, which can detect the blood perfusion in internal organs and tissues [13]. Recent evidence has found that DCE-MRI can accurately evaluate the microcirculation level in tumor tissues by quantitatively evaluating the nature of blood vessels in tissues [14]. However, it remains undefined whether DCE-MRI imaging parameters were of clinical value in the early diagnosis of HCC.

Accordingly, this study mainly explored the early diagnostic value of DCE-MRI hemodynamic parameters in patients with HCC, providing auxiliary reference for clinical diagnosis of HCC.

## 2. Methods and Materials

### 2.1. Clinical Data

A retrospective study was conducted on 50 HCC patients (patient group, PG) diagnosed in our hospital from May 2017 to May 2020, including 30 male patients and 20 female patients, with an average age of 56.7 ± 6.4 years. Concurrently, 45 patients with benign liver nodules diagnosed in our hospital were selected (control group, CG), including 27 male patients and 18 female patients, with a mean age of 55.2 ± 4.9 years. The Hospital Medical Ethics Committee approved the study protocol without reserves. Inclusion criteria for PG are as follows: HCC diagnosis by pathological examination; first diagnosis; and meeting *the Guidelines for Diagnosis and Treatment of Primary Liver Cancer in China (2017 Edition)* [15]. Exclusion criteria for PG are as follows: other tumors; serious infectious diseases, infectious diseases, and immune system diseases; and inability to perform DCE-MRI with related contraindications. Inclusion criteria for CG are as follows: treatment-naive patients with first diagnosis of benign liver nodules based on clinical manifestations and histopathological examination. Exclusion criteria for CG are as follows: other tumors; serious infectious diseases, infectious diseases, and immune system diseases; and inability to perform DCE-MRI with related contraindications. This study was reviewed by the Xi'an Central Hospital Ethics Committee, and all patients gave informed consent (no. XA977-832).

### 2.2. Detection Methods and Data Processing

The Siemens Magnetom Avanto 3.0T imaging system was used to examine the patient in a prone position with palms down. The abdominal phased array coil was set to 6 channels, and the abdominal belt was used to adjust the abdominal breathing of patients. After conventional plain scanning, dynamic, rapid, and enhanced scans were performed, with the scanning parameters set as field of view (FOV) = 320 × 290 mm, TE = 1.4 ms, TR = 4.1 ms, layer spacing = 0, layer thickness = 3.6 mm, matrix = 290 × 185, and excitation times = 1. During enhanced scanning, only the flip angle (3°, 9°, and 25°) was adjusted, and a total of 50 dynamic cycles (6 s/cycle) were scanned, with the entire scan lasting for about 5 min. Dynamic enhanced scanning was performed after the lesions were accurately located, and contrast agent GD-DTPA was injected intravenously with a high-pressure syringe at 0.1 mmol/kg and an injection flow rate of 2.5 mL/s. Subsequently, 15 mL isotonic saline was injected at the same flow rate to flush the pipeline. Patients were required to hold their breath during the whole dynamic enhanced scan, and only slight and rapid ventilation could be performed. The transfer coefficient (*K*^trans^) from intravascular to extracellular space and the rate constant (*K*_ep_) from extracellular space to intravascular transport were measured in three fields of inte. The DCE-MRI images were postprocessed using the magnetic resonance instrument supporting workstation (Siemens Tissue4D). The first step is to correct respiratory artifacts, calculate the T1 value, and then select appropriate parameters for measuring the lesion area and normal area, including, *K*^trans^, *K*_ep_, Ve, and iAUC.

### 2.3. Outcome Measures

Primary outcome measures are as follows: *K*^trans^, *K*_ep_, and AFP expression were compared between cases and controls. The diagnostic value of *K*^trans^ and *K*_ep_ in HCC was observed.

Secondary outcome measures are as follows: the correlation of *K*^trans^ and *K*_ep_ with patient clinical data was analyzed. The diagnostic value of either *K*^trans^ or *K*_ep_ combined with AFP in clinical staging, differentiation, and distant metastasis was compared.

### 2.4. Statistical Analysis

The software used for data analysis and image rendering was SPSS20.00 and GraphPad 8, respectively. K-S test was used to analyze the data distribution, in which *t* test was used for normal distribution data, and independent sample *t* test was used for intergroup comparison. The categorical data, which were given percentages (%), were analyzed using the chi-square test (*X*^2^). Logistic regression was used for joint prediction, and the receiver operating characteristic (ROC) curves were drawn to visualize the diagnostic value of imaging parameters and AFP in patients with primary HCC. A significance level of *P* < 0.05 was used in all analyses.

## 3. Results

### 3.1. *K*^trans^, *K*_ep_, and AFP Expression in Patients

**Figure 1:**
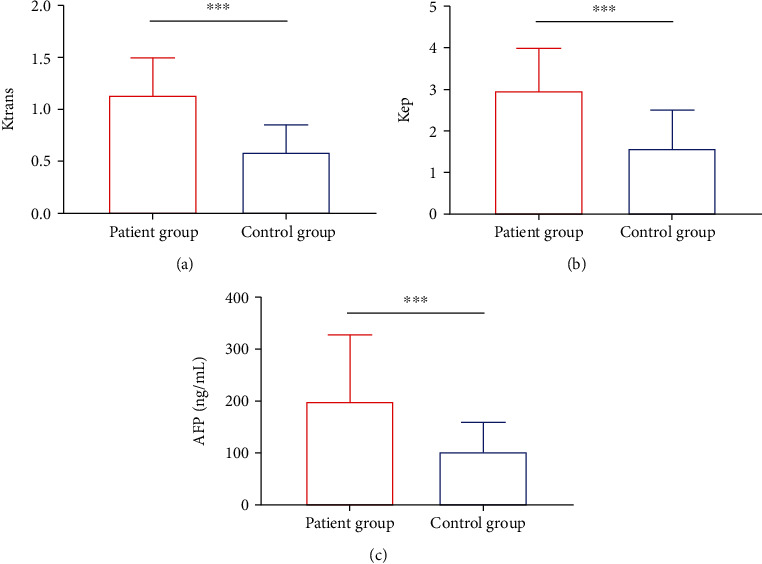
Expression of *K*^trans^, *K*_ep_, and AFP in patients. (a) Comparison of *K*^trans^ between control group and patient group. (b) Comparison of *K*_ep_ between control group and patient group. (c) Comparison of AFP level between control group and patient group. ^∗∗∗^*P* < 0.001.

We first analyzed *K*^trans^, *K*_ep_, and AFP expression in patients. Statistics revealed that *K*^trans^, *K*_ep_, and AFP were significantly higher in cases than in controls, with statistical differences (*P* < 0.05, Figures 1(a)–1(c)).

### 3.2. Diagnostic Value of *K*^trans^ and *K*_ep_ in HCC

**Figure 2:**
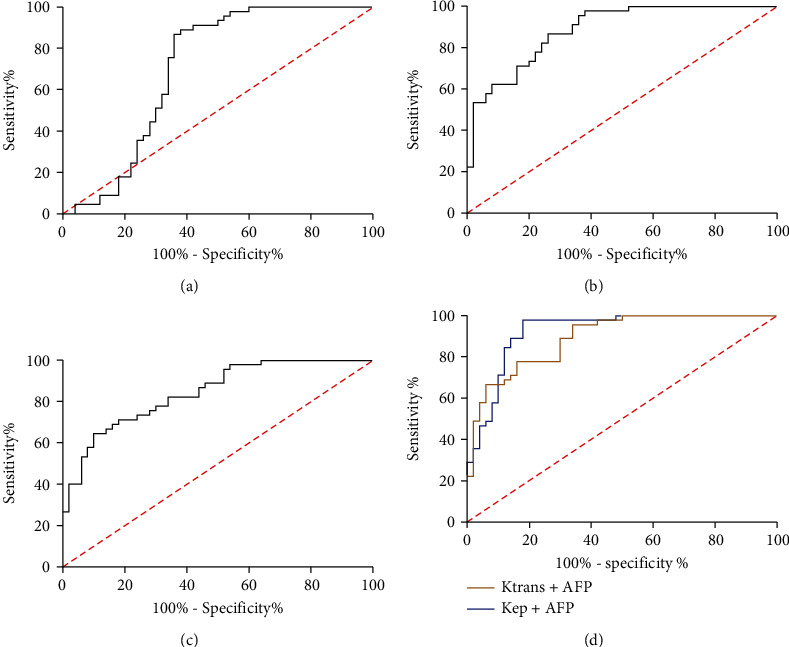
Diagnostic value of *K*^trans^, *K*_ep_, and AFP in HCC. (a) The AUC of AFP in the diagnosis HCC and benign nodules. (b) The AUC of *K*^trans^ in the diagnosis of HCC and benign nodules. (c) The AUC of *K*_ep_ in the diagnosis HCC and benign nodules. (d) The AUC of *K*^trans^+AFP and *K*_ep_+AFP in the diagnosis of HCC and benign nodules.

ROC curves for HCC diagnosis were drawn according to *K*^trans^, *K*_ep_ and AFP levels in patients. The results showed that the area under the curve (AUC) of AFP in distinguishing HCC patients from benign nodules was 0.703, with specificity of 62.00% and sensitivity of 88.89%, while the AUC, specificity, and sensitivity of *K*^trans^ were 0.885, 74.00%, and 86.67%, and those of *K*_ep_ were 0.844, 90.00%, and 64.44%, respectively. The joint analysis showed that the AUC of *K*^trans^+AFP was 0.923, the specificity was 82.00, and the sensitivity was 97.78, while the AUC of *K*_ep_+AFP was 0.892, the specificity was 77.78, and the sensitivity was 84.00. Therefore, the combined diagnosis with either *K*^trans^ or *K*_ep_ can improve the specificity of AFP in benign nodules (Figures 2(a)–2(d)).

### 3.3. Correlation of *K*^trans^, *K*_ep_, and AFP with Patient Clinical Data

To further understand the clinical value of *K*^trans^ and *K*_ep_ in HCC patients, we analyzed their correlations with patient clinical data. Through analysis, we found that *K*^trans^ and *K*_ep_ presented elevated expression in HCC patients with clinical stage III-IV, low differentiation, and distant metastasis (*P* < 0.05, Table 1).

### 3.4. Diagnostic Value of *K*^trans^ and *K*_ep_ Combined with AFP in Clinical Staging

**Figure 3:**
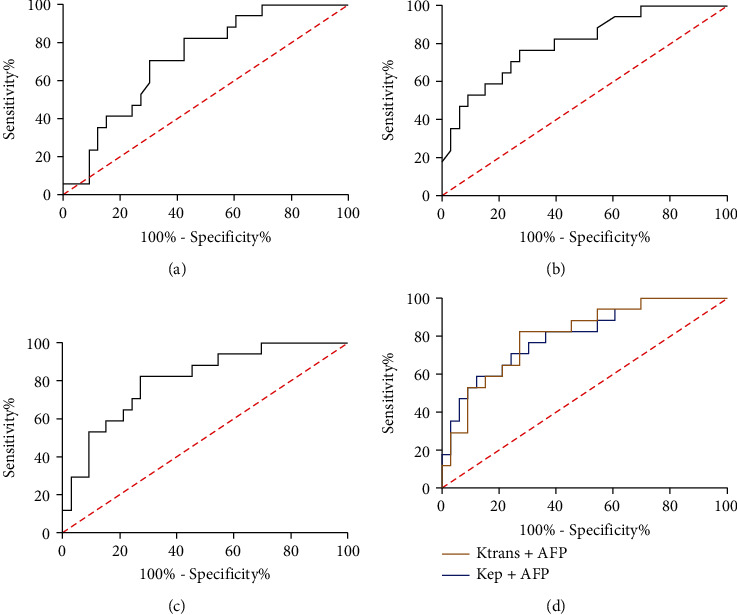
Diagnostic value of *K*^trans^ and *K*_ep_ combined with AFP in HCC clinical staging. (a) The AUC of AFP in diagnosing HCC and HCC clinical stages. (b) The AUC of *K*^trans^ in diagnosing HCC and HCC clinical stages. (c) The AUC of *K*_ep_ in diagnosing HCC and HCC clinical stages. (d) The AUC of *K*^trans^+AFP and *K*_ep_+AFP in diagnosing HCC and HCC clinical stages.

We plotted the ROC curves for the diagnosis of HCC based on *K*^trans^, *K*_ep_, and AFP expression. It was found that the AUC of AFP in distinguishing stage I-II from stage III-IV patients was 0.715, with a specificity of 69.69% and a sensitivity of 70.58%, while the AUC of *K*^trans^ and *K*_ep_ in distinguishing patients with HCC and those with benign nodules was 0.801 and 0.806, with specificity of 72.72% and 76.47% and sensitivity of 72.72% and 82.35%, respectively. The AUC of *K*^trans^+AFP was 0.800, the specificity was 87.87%, and the sensitivity was 58.83%, while the AUC of K_ep_+AFP curve was 0.804, the specificity was 72.72%, and the sensitivity was 82.35%. Therefore, the combined diagnosis with either *K*^trans^ or *K*_ep_ can improve the specificity and sensitivity of AFP in distinguishing HCC clinical staging (Figures 3(a)–3(d)).

### 3.5. Diagnostic Value of *K*^trans^ and *K*_ep_ Combined with AFP in Differentiation Degree

**Figure 4:**
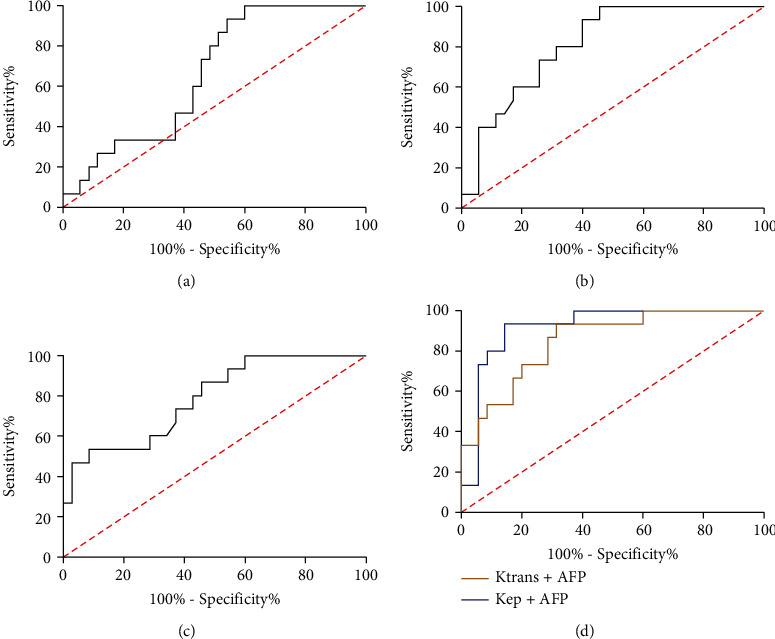
Diagnostic value of *K*^trans^ and *K*_ep_ combined with AFP in HCC differentiation. (a) The AUC of AFP in diagnosing HCC and HCC differentiation. (b) The AUC of *K*^trans^ in diagnosing HCC and HCC differentiation. (c) The AUC of *K*_ep_ in diagnosing HCC and HCC differentiation. (d) The AUC of *K*^trans^+AFP and *K*_ep_+AFP in diagnosing HCC and HCC differentiation.

ROC curves for HCC diagnosis were drawn based on *K*^trans^, *K*_ep_, and AFP in patients. The results showed that the AUC, specificity, and sensitivity of AFP were 0.661, 40.00%, and 100.00% in patients with different degrees of differentiation. The AUCs of *K*^trans^ and *K*_ep_ in HCC patients and controls with benign nodules were 0.813 and 0.785, with specificity of 54.28% and 91.42% and sensitivity of 100.00% and 53.33%, respectively. The analysis of joint detection showed that the AUC, specificity, and sensitivity of *K*^trans^+AFP were 0.916, 85.71%, and 93.33%, respectively, while the AUC of *K*_ep_+AFP was 0.851, the specificity was 68.57%, and the sensitivity was 93.33%. Therefore, the combined diagnosis with either *K*^trans^ or *K*_ep_ can improve the specificity of AFP in identifying the differentiation degree of HCC (Figures 4(a)–4(d)).

### 3.6. Diagnostic Value of *K*^trans^, *K*_ep_, and AFP in Distant Metastasis

**Figure 5:**
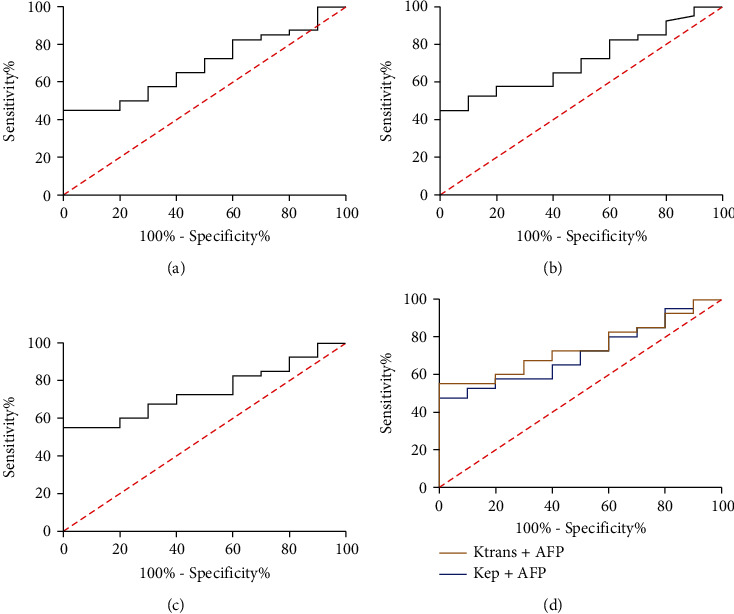
Diagnostic value of *K*^trans^, *K*_ep_ and AFP in HCC distant metastasis. (a) AUC of AFP in diagnosing HCC and HCC distant metastasis. (b) AUC of Ktrans in diagnosing HCC and HCC distant metastasis. (c) AUC of C. *K*_ep_ in diagnosing HCC and HCC distant metastasis. (d) AUC of *K*^trans^+AFP and *K*_ep_+AFP in diagnosing HCC and HCC distant metastasis.

According to *K*^trans^, *K*_ep_, and AFP expression in patients, ROC curves for HCC diagnosis were drawn. The results showed that AFP had an AUC of 0.690, a specificity of 100.00%, and a sensitivity of 45.45% in differentiating distant metastasis of HCC patients, whereas the AUCs of *K*^trans^ and *K*_ep_ in HCC patients and benign nodules were 0.711 and 0.742, with specificity of 100.00% and 100.00% and sensitivity of 45.45% and 55.55%, respectively. Through joint analysis, it is found that the AUC, specificity, and sensitivity of *K*^trans^+AFP were 0.712, 100.00%, and 47.50%, respectively, while the AUC of *K*_ep_+AFP was 0.742, the specificity was 100.00%, and the sensitivity was 55.55%. Therefore, the sensitivity of *K*^trans^ or *K*_ep_ combined with AFP in distant metastasis has not been significantly improved (Figures 5(a)–5(d)).

### 3.7. Pathological Images

**Figure 6:**
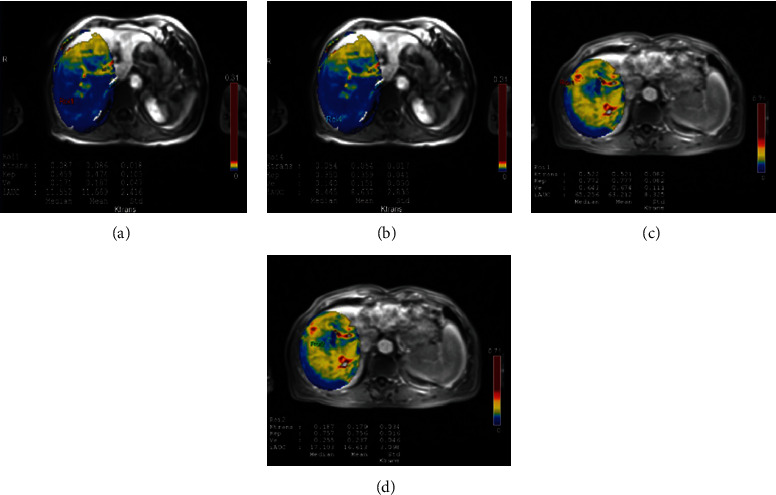
Hepatic cancer and paracarcinoma tissue, benign liver lesions, and normal liver tissue around benign liver lesions. Note: (a) Quantitative parameters of hepatic hemangioma; (b) quantitative parameters of normal liver tissue around hepatic hemangioma; (c) quantitative parameters of hepatocellular carcinoma; and (d) quantitative parameters of normal liver tissue around hepatocellular carcinoma.

HCC showed high blood perfusion on DCE-MRI pseudocolor images, and normal liver tissue showed low blood perfusion (Figures 6(a)–6(d)).

## 4. Discussion

At present, the pathogenesis of HCC remains elusive [16]. The existing academic research shows that HCC, characterized by high degree of malignancy, strong infiltration, and metastasis, is the result of synergistic effects of multiple factors, such as viral hepatitis, liver cirrhosis, aflatoxin pollution, and genetic factors. The prognosis of patients depends on whether early diagnosis and early treatment can be carried out [17, 18].

AFP is a glycoprotein primarily synthesized in fetal liver, and its increased expression in adults is mainly found in various malignancies such as liver cancer, gastric cancer and lung cancer. AFP is a common marker for early auxiliary diagnosis of liver cancer, but its sensitivity and specificity are low when detected alone [19, 20]. With the continuous development of imaging, the diagnostic value of ultrasound, CT, and MRI in HCC is increasingly concerned. Among them, MRI has become one of the mainstays for diagnosis and follow-up after treatment of HCC [21, 22]. DCE-MRI is a noninvasive imaging technique, the principle of which is that tissue T1 is shortened after intravascular injection of paramagnetic contrast agent. If repeated imaging is performed, the change of signal intensity in tissues can be measured, and the diffusion of contrast agent to surrounding tissues can be monitored over time [23]. DCE-MRI technology, different from traditional MRI, has developed from simple imaging anatomical morphological examination to micromolecular examination, with higher objectivity and accuracy [6]. The principle of the quantitative method of DCE-MRI is usually to use the blood flow double-chamber dynamics model, which is a mathematical analysis of the curve trend of the known drug molecular dynamics model [7]. The commonly used parameters *K*^trans^, *K*_ep_, *Ve*, and iAUC can reflect the transport status of contrast agents in the intravascular space and extravascular extracellular space, while the microenvironment of benign hepatic mass and HCC liver tissue has changed, vascular integrity, permeability pressure, etc. will also change accordingly, and the transport state of the contrast agent will be significantly different, which is manifested by the differences in *K*^trans^, *K*_ep_, Ve, and iAUC [7, 8]. Therefore, it has important clinical significance in tumor diagnosis and prognosis evaluation. Studies have shown that DCE-MRI has been well applied in prostate cancer, breast cancer, lung cancer, and other malignant tumors [24, 25]. In this paper, we found notably elevated *K*^trans^ and *K*_ep_ in HCC patients than in patients with benign nodules. Wang et al. [26] also found that *K*^trans^ and *K*_ep_ were highly expressed in HCC patients, which was consistent with our research results. After further analysis, we found that the AUC of either *K*^trans^ or *K*_ep_ combined with AFP detection was >0.8, which indicated that the combined detection had higher clinical value and could enhance the specificity of AFP in diagnosing HCC. We speculate that this is mainly because tumor progression depends on the generation of tumor blood vessels, and DCE-MRI can accurately distinguish HCC from liver metastases by effectively reflecting the hemodynamic changes.

Furthermore, we analyzed the relationship between *K*^trans^, *K*_ep_, and clinical data of HCC patients. Through analysis, we found that *K*^trans^ and *K*_ep_ in HCC patients with III+IV stage, low differentiation, and distant metastasis increased significantly, which indicated that *K*^trans^ and *K*_ep_ might have potential value in the diagnosis of HCC patients. Therefore, ROC curves were drawn based on different clinical data. It was identified that *K*^trans^ and *K*_ep_ alone did have high clinical value in the clinical staging and differentiation of HCC patients, both of which can improve the specificity and sensitivity of AFP in the clinical staging and differentiation of HCC patients through combined detection. We believe that HCC is a primary tumor accompanied by neovascularization. *K*^trans^ and *K*_ep_ mainly reflect the blood volume and distribution in blood vessels and are related to the total amount of blood perfusion in tissues, the surface area of blood vessels, and the permeability of blood vessels [27]. However, in the case of low tissue capillary permeability, the contrast dose through the endothelium is limited by vascular permeability. At this time, *K*^trans^ is equal to the product of permeability surface area per unit volume of tissue, and the higher the *K*^trans^ is, the higher the malignant degree of the tumor is [28, 29]. What is more, our study found that *K*^trans^ and *K*_ep_ were of low diagnostic value in HCC patients with distant metastasis, and the detection rate of AFP in distant metastasis was not greatly improved by the combined detection.

Herein, we determined through analysis that the DCE-MRI parameters *K*^trans^ and *K*_ep_ were of favorable diagnostic value in HCC. However, there is still room for improvement in this study. For instance, the number of samples included is small, and the results of this study as a retrospective study may be biased. Therefore, we hope that in the follow-up study, we can verify our research results by collecting more clinical samples and conducting a large sample cohort study.

## 5. Conclusion

DCE-MRI hemodynamic parameters *K*^trans^ and *K*_ep_ have high clinical value in early diagnosis and differentiation of HCC patients. The limitation of this study should be highlighted. Due to the abundant blood vessels and high permeability of HCC tissue, the amount of contrast agent entering the tumor from blood vessels is also higher than that of normal liver tissue, and the iAUC value increases, while the value of Ve is related to cell density and vascular permeability, which makes Ve have a certain uncertainty in the value that has not been tested in this study. Hence, ongoing trials including in this indicator are warranted.

## Figures and Tables

**Table 1:** Relationship between *K*^trans^, *K*_ep_, and clinical data of patients.

Variables	*K* ^trans^	*K*_ep_	AFP (ng/mL)
Age			
≥ 55 years old (*n* = 27)	1.09 ± 0.34	2.86 ± 0.98	187.98 ± 121.88
< 55 years old (*n* = 23)	1.00 ± 0.28	2.61 ± 0.80	159.19 ± 107.87
Gender			
Male (*n* = 30)	1.15 ± 0.35	3.01 ± 0.99	205.06 ± 125.01
Female (*n* = 20)	1.11 ± 0.39	2.90 ± 1.03	190.60 ± 134.59
Tumor size			
≥ 3 cm (*n* = 18)	1.17 ± 0.40	3.06 ± 1.04	216.64 ± 131.33
< 3 cm (*n* = 32)	1.11 ± 0.35	2.94 ± 0.99	189.51 ± 126.77
Clinical staging			
I-II (*n* = 33)	1.01 ± 0.30	2.63 ± 0.91	166.07 ± 124.57
III-IV (*n* = 17)	1.38 ± 0.35^∗^	3.67 ± 0.81^∗^	262.14 ± 110.60^∗^
Differentiation degree			
Medium+high differentiation (*n* = 35)	1.05 ± 0.35	2.76 ± 1.02	169.28 ± 127.64
Low differentiation (*n* = 15)	1.33 ± 0.31^∗^	3.51 ± 0.75^∗^	269.27 ± 100.35^∗^
Distant metastasis			
With (*n* = 10)	1.38 ± 0.32	3.61 ± 0.73	288.62 ± 94.29
Without (*n* = 40)	1.07 ± 0.35^∗^	2.83 ± 1.01^∗^	176.94 ± 126.09^∗^

∗ indicates *P* < 0.05 compared with the data in the group.

## Data Availability

The datasets used during the present study are available from the corresponding author upon reasonable request.
